# Diagnosis of Dementia and Cognitive Impairment

**DOI:** 10.3390/diagnostics9040180

**Published:** 2019-11-07

**Authors:** Andrew J. Larner

**Affiliations:** Cognitive Function Clinic, Walton Centre for Neurology and Neurosurgery, Lower Lane, Fazakerley, Liverpool L9 7LJ, UK; a.larner@thewaltoncentre.nhs.uk

**Keywords:** dementia, diagnosis, mild cognitive impairment

## Abstract

In this special issue of *Diagnostics*, expert contributors have produced up-to-date research studies and reviews on various topics related to the diagnosis of dementia and cognitive impairment. The methods of the assessments discussed extend from simple neurological signs, which may be elicited in the clinical encounter, through cognitive screening instruments, to sophisticated analyses of neuroimaging and cerebrospinal fluid biomarkers of disease. It is hoped that these various methods may facilitate earlier diagnosis of dementia and its subtypes, and provide differential diagnosis of depression and functional cognitive disorders, as a prelude to meaningful interventions.

Dementia and cognitive impairment represent one of the most pressing medical issues of our time, an issue that is likely to escalate as the world population ages. The consequences of this evolving demography pose huge challenges, from the clinical, social, and economic perspectives. Hence, this special issue devoted to the “Diagnosis of Dementia and Cognitive Impairment”.

From the clinical standpoint, diagnosis of dementia and cognitive impairment is often the starting point for intervention. The need to identify individuals with dementia and cognitive impairment in order to initiate appropriate treatment and management options is self-evident, yet it remains the case that many afflicted individuals remain undiagnosed, the so-called “dementia diagnosis gap”. However, increasing societal awareness of dementia may have prompted an increased frequency of presentation of individuals with purely subjective memory complaints, making functional cognitive disorders an important differential diagnosis of dementia and cognitive impairment.

Various methods to facilitate the accurate diagnosis of dementia and cognitive impairment may be used. These include neurological history taking and clinical examination for particular signs, the administration of cognitive screening instruments, various neuroimaging techniques, and the examination of cerebrospinal fluid for disease biomarkers. All are addressed in this volume.

History taking, both anamnesis, and heteroamnesis, is central to initial patient assessment when either mild cognitive impairment (MCI) or dementia is suspected. This is supplemented by the neurological examination, wherein certain signs may point to the diagnosis. These include both traditional (“canonical”) textbook neurological signs and more recently investigated (“non-canonical”) signs, such as the attended alone sign, the head-turning sign, and the applause sign [1]. In this issue, Professor Ahmet Turan Isik and his colleagues, who have previously combined these signs as the “Triple Test” [2], show their utility in combination with a cognitive screening instrument, the Rapid Cognitive Screening Test, for the assessment of older adults [3].

Cognitive screening instruments using pencil and paper tests, which are easily applicable at the clinical level, continue to be a mainstay of assessment for cognitive impairment and dementia, particularly in resource-poor or resource-limited settings. There is no shortage of such tests [4], all with their potential advantages and shortcomings. Jerry Brown and his colleagues present an update on the Test Your Memory (TYM) test, which is self-administered under supervision, and the TYM-MCI (formerly Hard-TYM) for the diagnosis of amnestic MCI and mild AD, which is clinician-administered [5]. New data on the use of TYM in a general neurology clinic and as a telephone-administered test are presented, suggesting potential value in these settings [6]. Hartmut Lehfeld and Mark Stemmler demonstrate the potential utility of the Syndrom Kurtztest (SKT), a short cognitive performance test addressing memory and attention, the latter related to the speed of information processing [7]. Ronan O’Caoimh and William Molloy compare the standardised Mini-Mental State Examination (sMMSE) to the Quick Mild Cognitive Impairment (Qmci) screen, finding the latter to have similar or greater accuracy in distinguishing all dementia subtypes and particularly MCI [8]. I present data from a large pragmatic study of the Mini-Addenbrooke’s Cognitive Examination (MACE) [9,10], and with Besa Ziso describe attempts to modify the Cognitive Disorders Examination (Codex) [11] to improve detection of MCI [12].

Although routinely and widely used, pencil and paper, cognitive screening tests have many shortcomings. Emma Elliott, Terry Quinn, and their colleagues identify factors associated with full or partial incompletion of cognitive screeners in the context of stroke, finding that around a quarter of patients were fully untestable. The clock drawing test proved the most incomplete of the tests [13]. These issues of test feasibility and how best to handle incomplete data remain to be resolved [14]. Some of the problems with pencil and paper cognitive screening tests might be circumvented by using computerised testing batteries. Reviewing this approach, Avital Sternin, Alistair Burns, and Adrian Owen note advantages such as personalised assessment, at-home testing, measurement of response times, and interpretation of test scores in the context of extensive normative data, permitting the use of “meaningful change” and “validity” indices [15].

Disease biomarkers may be characterised into various subtypes, including diagnostic, predictive, monitoring, and prognostic. Hitherto largely tools of research, biomarkers are now incorporated into diagnostic criteria for neurodegenerative disorders (e.g., Alzheimer’s disease [16,17], dementia with Lewy bodies [18]) and their assessment is increasingly becoming part of day-to-day clinical practice. A comprehensive overview of the use of amyloid positron emission tomography (PET) in Alzheimer’s disease is provided here by Subapriya Suppiah, Mellanie-Anne Didier, and Sobhan Vinjamuri [19]. Cerebrospinal fluid (CSF) biomarkers have been extensively studied by Miguel Tábuas-Pereira and his colleagues, for example, in connection with the head-turning sign [20] and seizures in Alzheimer’s disease [21]. Herein, they suggest a novel potential role for CSF amyloid as a prognostic biomarker for reduced survival in a cohort of patients with frontotemporal dementia [22].

Dementia and cognitive impairment have a broad differential diagnosis [23]. In clinical practice, one of the most challenging differentials is depression, which may be both a risk factor for, and comorbid with, dementia. This interaction is illustrated by O’Caoimh and Molloy, who found that patients with both dementia and depression scored higher on cognitive screening tests (sMMSE and Qmci) than those with dementia only, whereas patients with both MCI and depression scored lower on these tests than those with MCI only. Hence, comorbid depression lowered the diagnostic accuracy of sMMSE and Qmci for dementia, but improved accuracy in those with MCI. O’Caoimh and Molloy suggest that comorbid depression may influence performance on cognitive testing in different ways depending on the stage of cognitive impairment, more so at earlier stages, perhaps increasing the risk of conversion to dementia [8]. Lehfeld and Stemmler show how the subtests of the SKT, examining memory and attention (speed of information processing), may assist in the differential diagnosis of depression versus MCI/mild dementia [7].

Another differential to be considered when assessing patients for dementia and cognitive impairment, and of increasing clinical relevance, is a functional cognitive disorder (FCD) [24]. Catherine Pennington, Harriet Ball, and Marta Swirski report their findings in a series of patients with FCD, noting an equivalent burden of cognitive symptomatology to MCI patients and similar impairment on a cognitive screening instrument (Montreal Cognitive Assessment) [25], as has also been noted with the MACE [26]. Disturbances of mood and sleep may be contributory factors to FCD [26]. Using the large datasets generated by online testing, Sternin, Burns, and Owen note that whilst older people sleep less, the amount of sleep required for optimal cognitive performance remains constant regardless of age, suggesting that more sleep may be a good idea as we age [15].

As this brief overview shows, the primary aims of this special issue have been to present information on promising new approaches to diagnose dementia and cognitive impairment, which are readily applicable to clinical practice and which also assist in the significant differential diagnosis of functional cognitive disorders. But what of the future? The dismal outcomes of clinical therapeutic trials for Alzheimer’s disease in recent years (which contrast so starkly with the great increase in understanding of the biology of the disease [27]) have suggested that by the time of clinical diagnosis the degree of brain injury may be largely irreversible, thereby demanding a proactive preventative approach to maintain brain health rather than a reactive therapeutic response to palliate brain disease. How might the preclinical, presymptomatic phases of the disease, which are recognised to be of decades duration [28], be reliably and accurately identified, and hence amenable to the application of (yet to be identified) disease-modifying treatments?

One way forward may be suggested by a recent report finding accelerated long term forgetting to be a feature of presymptomatic stages of (autosomal dominant) Alzheimer’s disease [29]. Obviously, the finding requires independent corroboration, but if true, it might indicate one approach to the “bioprediction” of Alzheimer’s disease, perhaps in combination with amyloid PET imaging and CSF biomarkers and genetic panels examining risk factor alleles. The bioprediction approach seeks to conceptualise medical “disorder” in terms of probabilistic modelling, based on present and future risks of harm rather on a binary or categorical formulation (disorder or normalcy) and is justified by the belief that disease biomarkers will not map cleanly onto clinical diagnostic categories. “Risk banding”, based on the shape of a “probability dysfunction” model unique to each individual, might be used to determine the necessity or otherwise for response/intervention [30].

It has been a great honor and privilege for me to guest edit this timely special issue. I am grateful to all the authors for their willingness to contribute and for their prompt submission of manuscripts. I hope readers will derive as much interest and stimulus in reading these articles as I have in curating them.
